# Dissociating tinnitus patients from healthy controls using resting-state cyclicity analysis and clustering

**DOI:** 10.1162/netn_a_00053

**Published:** 2018-10-01

**Authors:** Benjamin J. Zimmerman, Ivan Abraham, Sara A. Schmidt, Yuliy Baryshnikov, Fatima T. Husain

**Affiliations:** Beckman Institute for Advanced Science & Technology, University of Illinois at Urbana-Champaign, IL, USA; Department of Speech and Hearing Science, University of Illinois at Urbana-Champaign, IL, USA; Department of Electrical & Computer Engineering, University of Illinois at Urbana-Champaign, IL, USA; Beckman Institute for Advanced Science & Technology, University of Illinois at Urbana-Champaign, IL, USA; Neuroscience Program, University of Illinois at Urbana-Champaign, IL, USA; Department of Electrical & Computer Engineering, University of Illinois at Urbana-Champaign, IL, USA; Department of Mathematics, University of Illinois at Urbana-Champaign, IL, USA; Beckman Institute for Advanced Science & Technology, University of Illinois at Urbana-Champaign, IL, USA; Department of Speech and Hearing Science, University of Illinois at Urbana-Champaign, IL, USA; Neuroscience Program, University of Illinois at Urbana-Champaign, IL, USA

**Keywords:** Tinnitus, Resting-state fMRI, Cyclicity, Classification

## Abstract

Chronic tinnitus is a common and sometimes debilitating condition that lacks scientific consensus on physiological models of how the condition arises as well as any known cure. In this study, we applied a novel cyclicity analysis, which studies patterns of leader-follower relationships between two signals, to resting-state functional magnetic resonance imaging (rs-fMRI) data of brain regions acquired from subjects with and without tinnitus. Using the output from the cyclicity analysis, we were able to differentiate between these two groups with 58–67% accuracy by using a partial least squares discriminant analysis. Stability testing yielded a 70% classification accuracy for identifying individual subjects’ data across sessions 1 week apart. Additional analysis revealed that the pairs of brain regions that contributed most to the dissociation between tinnitus and controls were those connected to the amygdala. In the controls, there were consistent temporal patterns across frontal, parietal, and limbic regions and amygdalar activity, whereas in tinnitus subjects, this pattern was much more variable. Our findings demonstrate a proof-of-principle for the use of cyclicity analysis of rs-fMRI data to better understand functional brain connectivity and to use it as a tool for the differentiation of patients and controls who may differ on specific traits.

## Author Summary

Chronic tinnitus is a common, yet poorly understood, condition without a known cure. Understanding differences in the functioning of brains of tinnitus patients and controls may lead to better knowledge regarding the physiology of the condition and to subsequent treatments. There are many ways to characterize relationships between neural activity in different parts of the brain. Here, we apply a novel method, called cyclicity analysis, to functional MRI data obtained from tinnitus patients and controls over a period of wakeful rest. Cyclicity analysis lends itself to interpretation as analysis of temporal orderings between elements of time-series data; it is distinct from methods like periodicity analysis or time correlation analysis in that its theoretical underpinnings are invariant to changes in time scales of the generative process. In this proof-of-concept study, we use the feature generated from the cyclicity analysis of the fMRI data to investigate group level differences between tinnitus patients and controls. Our findings indicate that temporal ordering of regional brain activation is much more consistent in the control population than in tinnitus population. We also apply methods of classification from machine learning to differentiate between the two populations with moderate amount of success.

## INTRODUCTION

Chronic subjective tinnitus, the long-term perception of a sound with no external source, is a relatively common hearing disorder affecting 4–15% of the population (Møller, 2007). A portion of these individuals are extremely distressed by the percept, which exerts a significant debilitating effect on their lives. However, the diagnosis of tinnitus and the assessment of tinnitus distress are typically made through self-report questionnaires, and a consensus regarding psychophysiological models of tinnitus is lacking. Part of the reason for impaired progress in developing an understanding of the biology of the disorder has been the discrepant and often contradictory evidence in the neuroscientific literature. An improved understanding of the neural underpinnings of tinnitus would benefit both diagnosis of the disorder as well as the generation of therapies and perhaps the predictability of a therapy’s effectiveness at an individual level.

Tinnitus is a highly heterogeneous condition in terms of etiology, laterality of the percept, type of sound (e.g., tonal, modulating, broad-band), pitch, loudness, age, and nature of onset, duration, and severity. This heterogeneity has made the condition difficult to study using structural (sMRI) and functional magnetic resonance imaging (fMRI), because MRI studies typically include relatively small sample sizes (see Friston, Holmes, & Worsley, 1999). Controlling for all the variables that may contribute to the neural underpinnings of tinnitus is therefore a difficult statistical challenge, as is interpreting differences between samples with diverse parameters for subject recruitment. The brain regions implicated in tinnitus commonly differ across studies, which may be due in part to the heterogeneity of the condition. It remains unclear whether functional differences are capable of reliably distinguishing tinnitus patients from normal controls, or distinguishing subgroups of patients with tinnitus.

Tinnitus-related brain differences have been observed across many applications of MRI, including anatomical analysis, task-based functional imaging, and resting-state functional connectivity (rs-FC) analyses (Adjamian, Sereda, & Hall, 2009; Allan et al., 2016; Husain, 2016; Husain & Schmidt, 2014). Rs-FC analysis finds interregional correlations of spontaneous brain activity, which reliably organizes into coherent resting-state networks (RSNs) (Fox et al., 2005; Mantini, Perrucci, Del Gratta, Romani, & Corbetta, 2007; Raichle & Snyder, 2007; Shulman et al., 1997). Rs-FC is an interesting candidate to examine for a significant neural change accompanying a disorder because communication between brain regions may be altered in the absence of large morphological changes. In addition, in the normal population, replicability of rs-FC is high (Shehzad et al., 2009). The replicability is comparable to task-based fMRI (Mannfolk et al., 2011), but unlike task-based fMRI, the experimenter may be less concerned that an experiment failed because of poor experimental design, since there is no task. In fact, rs-FC has been shown to be altered across a number of neuropsychological conditions (Barkhof, Haller, & Rombouts, 2014), and there is recent evidence that Rs-FC is also altered in tinnitus (Husain & Schmidt, 2014). Rs-FC is also relevant in tinnitus because those with the chronic condition are presumably aware of their internal sound while being scanned, whereas the control groups do not have such an internal sound to which they can attend.

However, it is possible that the general correlations between regions of the brain do not drastically change within tinnitus, but rather that some dynamic, time-varying relationship between regions would be more sensitive to the disorder. Rather than depending on static correlations between regions over an entire time course of the resting-state task, we considered manipulating the temporal qualities of the resting-state data to look for time-dependent patterns of activity between regions.

Many classical mathematical and statistical tools have been useful in analyzing signals, which are periodic in time or which seek to identify time-varying correlations between multiple signals. However, the signal may also be cyclic, but aperiodic, where there may be a consistent temporal ordering between signals without a predictable period between cycles. Fluctuations in the brain’s functional topology has been an emerging and important area of study within cognitive neuroscience (H. Chen et al., 2013). An analysis of cyclic relationships between brain regions would therefore be useful to examine the possibility of consistent temporal ordering of the spontaneous activity of certain brain regions—a so called “leader-follower” structure. This lead matrix provides the strength of the temporal ordering between pairs of regions of interest (ROIs) included in the analysis. Looking at differences in this leader-follower structure between groups may reveal important insights into understanding changes in global functional organization, and may subsequently help in the classification between members of these groups. To this end, we employed a recently developed cyclicity analysis method (Baryshnikov & Schlafly, 2016) on a set of tinnitus patients (TIN) and normal hearing controls (NH) to determine whether the cyclic relationships between brain regions has the potential to help distinguish these two groups.

In the present study, cyclicity analysis was performed on a subset of resting-state fMRI data acquired as part of a larger, ongoing study to better understand neural mechanisms of tinnitus. Multiple methods of classification were explored after dimension reduction. These methods included quadratic and linear support vector machines (SVM), linear and quadratic discriminant analysis (DA), and partial least squares discriminant analysis (PLS-DA). Because cyclicity is a novel way to analyze fMRI data, many characteristics of the cyclicity analysis were additionally explored.

## RESULTS

### Cyclicity Analysis

**Figure 1.**
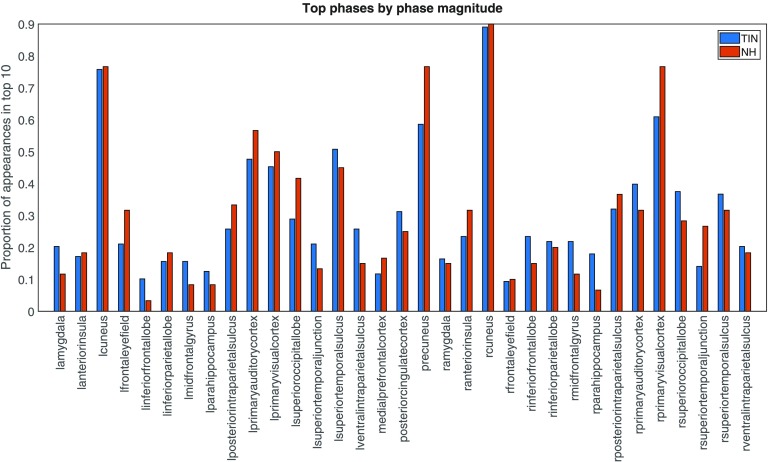
Regions with the highest magnitudes in the cyclicity analysis. The leading eigenvalue and the corresponding eigenvector of the lead matrix determine the magnitude; in particular, the elements of the eigenvector correspond to ROIs, and the larger an element’s modulus is, the greater the magnitude corresponding to the signal from that ROI. The chart shows the proportion of times each region occurred in the top 10 magnitudes for each subject. Bars are displayed for the tinnitus group and the normal hearing controls. This graph reveals that certain regions have consistently high magnitudes in the cyclicity analysis, especially in visual regions such as the right and left cuneus. This is true for both tinnitus and controls. In other regions, such as the precuneus, the phase magnitudes are more variable between groups.

The cyclicity analysis was performed on the time-series data from 33 ROIs for each subject, generating a lead matrix where each element of the matrix corresponds to the temporal ordering between two ROIs. The magnitude of the cyclic signal from an ROI can be analyzed by examining the leading eigenvalue and corresponding eigenvector of a subject’s lead matrix. A larger modulus of an element of that eigenvector translates into a larger signal corresponding to the ROI associated with that element. Greater magnitude cyclic signals here may be interpreted as a more constrained temporal ordering. The cyclicity analysis provides a cyclic ordering of ROIs, but the direction of the cycle cannot be determined. Therefore, the strength of the ordering, rather than the direction, is the focus of the analysis. The magnitude of the cyclic signals from each ROI were computed separately for both the controls and the tinnitus groups as in Figure 1. It was found that left and right cuneus corresponded to the greatest magnitude signals in both groups of subjects.

**Figure 2.**
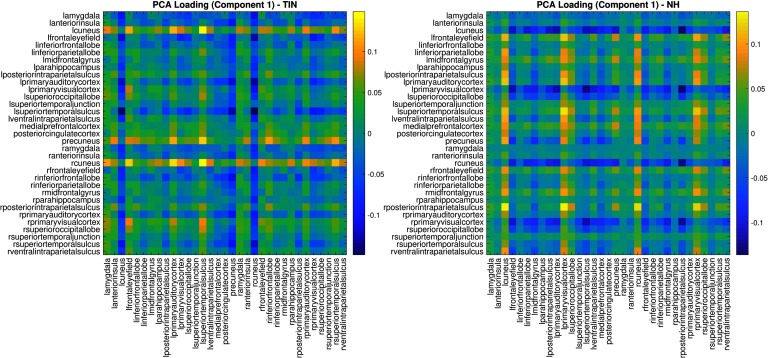
First principal component of each groups’ lead matrix. This figure shows the contribution of each ROI pair to the direction of greatest variation in each groups’ dataset. This is obtained from PCA and the first loading vector for each group is depicted here. The first component is interpreted as primarily representing cyclic connectivity with the left and right cuneus. Colors correspond to the unit normalized loadings. It is observed that for both the normal hearing controls and for tinnitus subjects, the ordering corresponding to the right and left cuneus is strongly determined, whereas the ordering between ROI pairs of other regions is much less evident.

Principal component analysis (PCA) was performed, and it was found further that the primary contributor to the direction of greatest variation in both test groups also corresponded to the left and right cuneus. This can be visualized in the matrix generated from the first component loading vector from the PCA analysis (Figure 2). The loading vector consists of coefficients in the linear combination of original variables that generate principal component scores. These findings together were interpreted to mean that the cuneus ROIs did not contribute any discriminatory information between the two groups. Therefore, these ROIs were removed from further analysis.

### Difference of First Principal Components Between Groups

**Figure 3.**
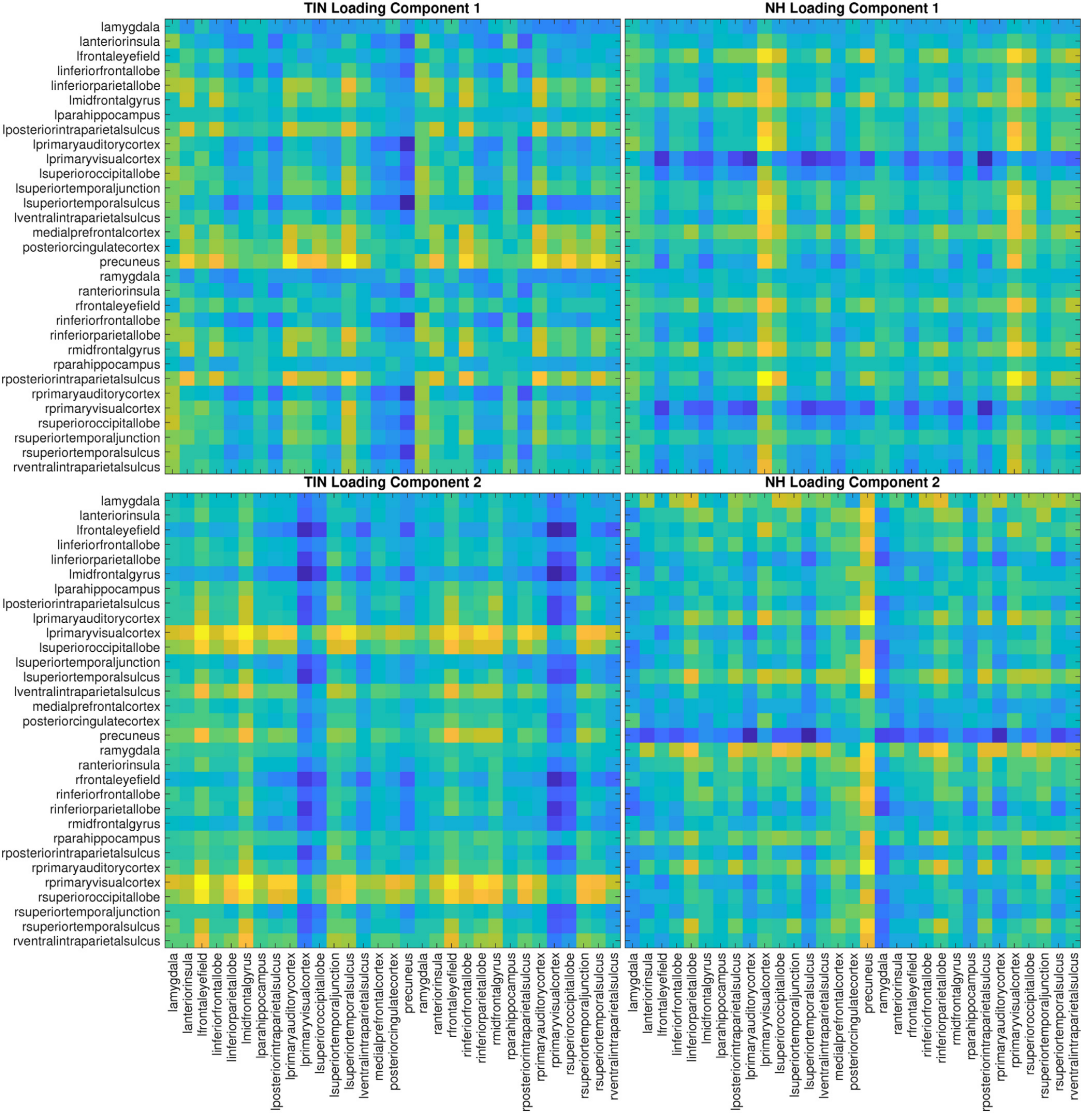
Differences in the first two components of the groups’ lead matrices. Similar to Figure 2, here we examine the first and second component loading vectors for tinnitus and normal hearing after removing the right and left cuneus from the cyclicity analysis. The first and second components appear to switch between the two groups. The eigenvalue ratios corresponding to the first two components, *λ*_1_/*λ*_2_, and the first and third components, *λ*_1_/*λ*_3_, for the TIN group are 1.51 and 2.51, respectively, while for NH group the same quantities are 1.97 and 2.84, respectively. Here *λ*_1_, *λ*_2_, *λ*_3_ are the leading eigenvalues of the covariance matrix of each group and therefore help quantify how much of the variance is explained by a particular component; a ratio of 1 would mean both components explain variance equally well.

The covariance structure of the data for each group was studied separately after removing the left and right cuneus from the time course data. PCA was used to achieve dimension reduction prior to classification. Figure 3 shows the first and second loading vectors (i.e., those that account for the most variance in the data) for the tinnitus and normal hearing controls. The first and second components appear to switch between the two groups. In the normal hearing controls, the first component is weighted toward the cyclic relationships with the primary visual cortices and occipital lobe, while the second component is more weighted toward the precuneus and amygdala. In contrast, in the tinnitus group, the first component seems to be strongly weighted toward the cyclic connectivity of the precuneus while the amgydala is less constrained, and the second component seems to be weighted toward the visual areas.

### Stability Analysis

**Figure 4.**
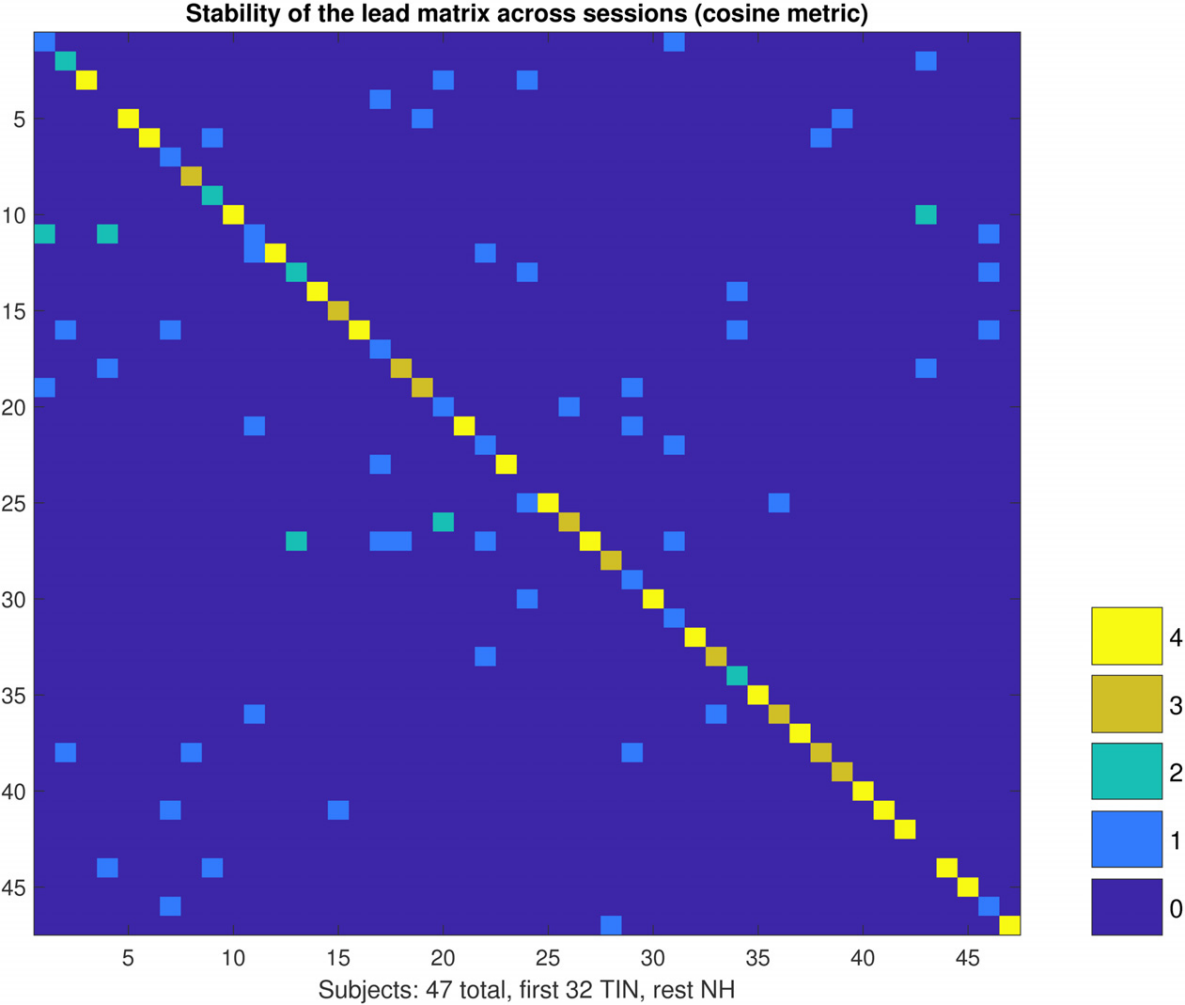
The stability of the lead matrix across sessions that were 1 week apart. Since the lead matrix is a feature constructed from the fMRI time series data, its consistency over time is investigated here. The figure above is a visualization of the confusion matrix arising from a classifier. Each row and column correspond to an individual subject in the analysis. The colors correspond to how many runs (out of 4 total runs) were correctly classified after training a 1-nearest neighbor classifier with the cosine metric on the other weeks data. The cosine metric serves as a measure of how closely aligned vectors are in high dimensional spaces. This graph shows good stability in the cyclic patterns of data, that is, consistent leader-follower relationships between ROIs, within subjects across 1 week.

The results of a *k*-nearest neighbor (*k*-NN) analysis for the subject classification across sessions is presented in Figure 4. In this figure, the row designates the subject. The goal of this analysis was to see if we could create a training set, based on the cyclicity analysis of data separated by 1 week from the testing set, and accurately predict if the cyclicity data from one run belonged to a specific subject. Perfect classification would mean that all the subjects were classified correctly four times (each run was classified correctly), and would look like uniform brightness down the diagonal of the figure. We found a 70% accuracy for classifying the runs, with 21 out of the 47 subjects being classified perfectly, signaling that the lead matrix is a viable candidate as a feature to use for classification.

### Traditional Classification

Four classification methods, linear SVM and DA, as well as quadratic SVM and DA methods were attempted after using PCA for dimension reduction to 10 features. To avoid overfitting or the curse of dimensionality only reduction up to 10 dimensions were tried for these methods. We also attempted PLS-DA using 20 latent components. The confusion matrices and accuracies from these analyses are shown in Table 1.

**Table 1.** Classification results

**Linear SVM**		
	Controls	Tinnitus
Controls	26.73%	73.27%
Tinnitus	19.38%	80.63%
Accuracy: 63.43%		

**Linear Discriminant**		
	Controls	Tinnitus
Controls	31.13%	68.87%
Tinnitus	20.75%	79.25%
Accuracy: 63.89%		

**Quadratic SVM**		
	Controls	Tinnitus
Controls	30.73%	69.27%
Tinnitus	27.00%	73.00%
Accuracy: 59.51%		

**Quadratic discriminant**		
	Controls	Tinnitus
Controls	15.87%	84.13%
Tinnitus	21.97%	78.03%
Accuracy: 58.81%		

**PLS-DA**		
	Controls	Tinnitus
Controls	32.45%	67.55%
Tinnitus	28.29%	71.71%
Accuracy: 59.03%		

*Note*. Rows represent the true class and columns represent the predicted class.

Because there was a greater number of tinnitus subjects in our sample than control subjects, we were interested in examining the same classification procedures after randomly selecting members from the tinnitus group to match the size of the normal hearing group before training a classifier. The results of this analysis are shown in Table 2.

**Table 2.** Classification results with equal class sizes

**Linear SVM**		
	Controls	Tinnitus
Controls	56.67%	43.33%
Tinnitus	42.93%	57.07%
Accuracy: 56.87%		

**Linear discriminant**		
	Controls	Tinnitus
Controls	56.47%	43.53%
Tinnitus	44.60%	55.40%
Accuracy: 55.93%		

**Quadratic SVM**		
	Controls	Tinnitus
Controls	54.20%	45.80%
Tinnitus	45.13%	54.87%
Accuracy: 54.53%		

**Quadratic discriminant**		
	Controls	Tinnitus
Controls	51.60%	48.40%
Tinnitus	48.47%	51.53%
Accuracy: 51.57%		

**PLS-DA**		
	Controls	Tinnitus
Controls	58.09%	41.91%
Tinnitus	40.77%	59.23%
Accuracy: 58.60%		

*Note*. Rows represent the true class and columns represent the predicted class.

### Wilks’ Lambda for Variable Selection

Wilks’ criterion was used to select variables, that is, elements of the lead matrices that were most discriminatory across the whole dataset. We selected the 20 most discriminating ROI pairs to use to reduce the dimensions of data in our training set instead of PCA. The classification results obtained by using data from only the top 20 most discriminating ROI pairs is presented in Table 3.

**Table 3.** Classification results using top 20 most discriminating ROI pairs

**Linear SVM**		
	Controls	Tinnitus
Controls	23.53%	76.47%
Tinnitus	17.25%	82.75%
Accuracy: 63.85%		

**Linear discriminant**		
	Controls	Tinnitus
Controls	34.07%	65.93%
Tinnitus	29.00%	71.00%
Accuracy: 59.21%		

**Quadratic SVM**		
	Controls	Tinnitus
Controls	32.93%	67.07%
Tinnitus	24.44%	75.56%
Accuracy: 61.96%		

**Quadratic discriminant**		
	Controls	Tinnitus
Controls	7.13%	92.87%
Tinnitus	7.81%	92.19%
Accuracy: 65.04%		

**PLS-DA**		
	Controls	Tinnitus
Controls	52.47%	47.53%
Tinnitus	31.31%	68.69%
Accuracy: 63.51%		

*Note*. Rows represent the true class and columns represent the predicted class.

**Figure 5.**
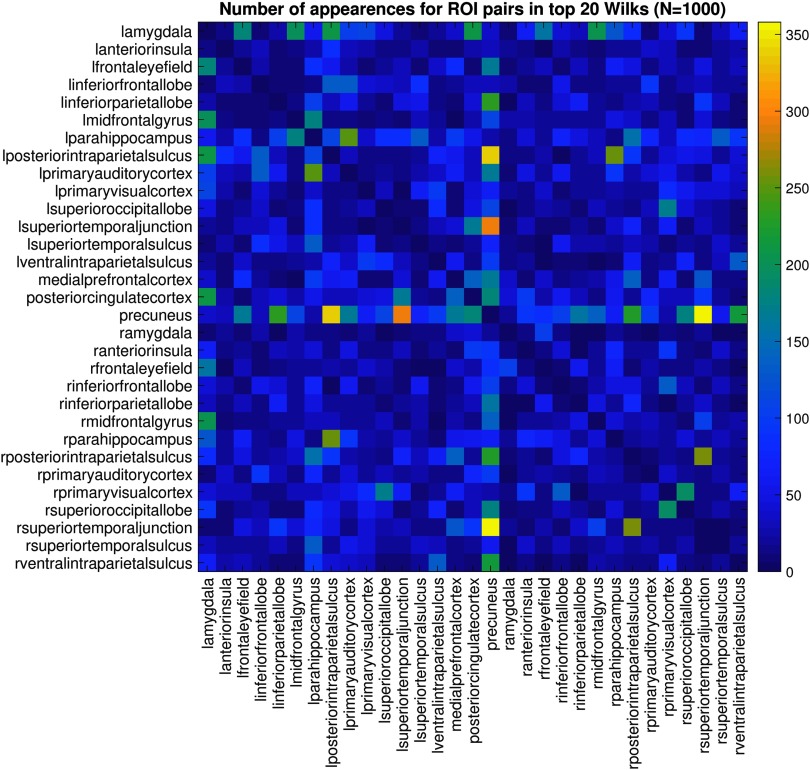
The most stable ROI pairs with respect to discriminatory ability across the dataset. The Wilks’ lambda criterion can be used to determine which features in the data have more discriminatory ability for classification. A thousand random and equally sized subsets of the data were examined, and the top 20 ranked ROI pairs (with respect to discriminatory ability) were recorded. The figure shows how many times an ROI pair appeared in the ranking.

Because some of these features were likely to be particularly discriminating in only this sample, it was of interest to further examine the stability of the ROI pairs selected by using the Wilks’ criterion. This was done by randomly selecting 1,000 subsets of the data and running Wilks analysis. The subset of ROI pairs that consistently showed up across a range of subsets is shown in Figure 5. We chose the top 10 most stable ROI pairs from this analysis to use for subsequent classification in order to compare with the results from the top 20 most discriminating ROI pairs (Table 4).

**Table 4.** Classification results using top 10 most stable discriminating ROI pairs

**Linear SVM**		
	Controls	Tinnitus
Controls	20.47%	79.53%
Tinnitus	11.25%	88.75%
Accuracy: 66.96%		

**Linear discriminant**		
	Controls	Tinnitus
Controls	30.73%	69.27%
Tinnitus	19.16%	80.84%
Accuracy: 64.85%		

**Quadratic SVM**		
	Controls	Tinnitus
Controls	31.73%	68.27%
Tinnitus	24.72%	75.28%
Accuracy: 61.38%		

**Quadratic discriminant**		
	Controls	Tinnitus
Controls	23.60%	76.40%
Tinnitus	27.91%	72.09%
Accuracy: 56.62%		

**PLS-DA**		
	Controls	Tinnitus
Controls	50.47%	49.53%
Tinnitus	25.20%	74.80%
Accuracy: 67.03%		

*Note*. Rows represent the true class and columns represent the predicted class.

### Examination of Elements Identified Through Wilks’ Lambda

**Figure 6.**
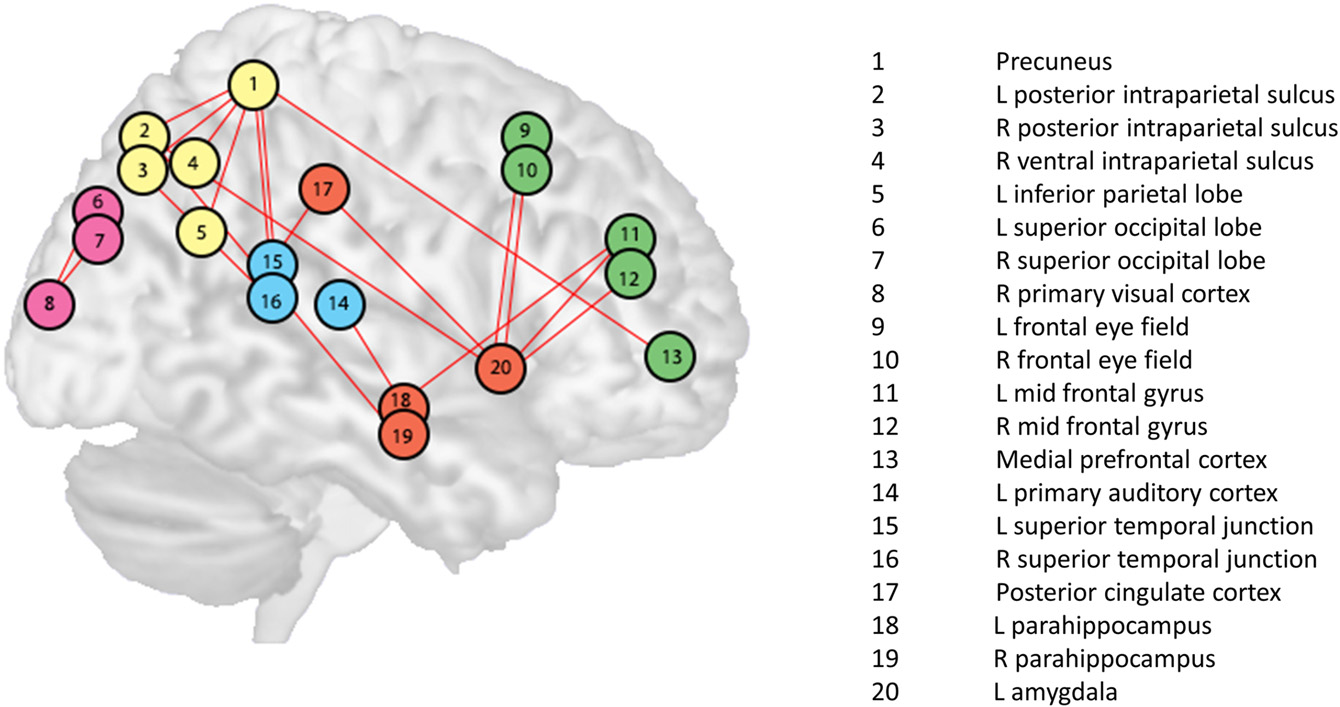
Graphical representation of the 20 most discriminating ROI pairs that help distinguish TIN from NH.

**Figure 7.**
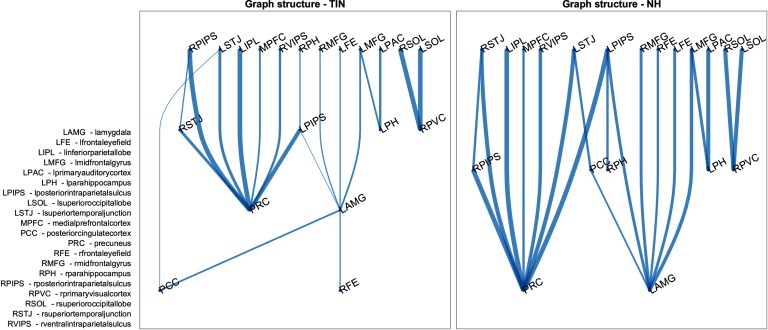
Graph structure of leader-follower connections. This figure shows the direction of leader-follower relationships between 20 ROI pairs that most help discriminate the normal hearing controls from the tinnitus subjects (activity follows downward). A node is assigned to each layer as soon as possible provided its predecessors have already appeared. The thickness of the edges corresponds to the proportion of subjects with that direction, and thus reveals the consistency of the leader-follower connections. In normal hearing subjects, there is more consistent cyclic connectivity with the amygdala than in the tinnitus subjects.

**Figure 8.**
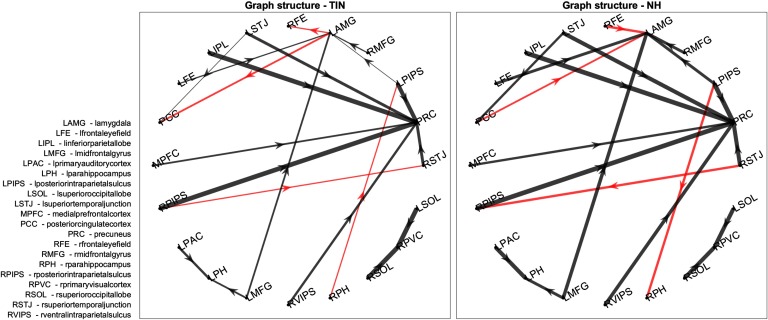
Differences in leader-follower direction between groups. This graph shows the same data as the previous figure, but fixes the node positions relative to each other for easy comparison and highlights the ROI pairs where the leader-follower relationship switches direction between the two groups.

The lambda values correspond to elements of the lead matrix, and thus to ROI pairs. The top 20 discriminatory ROI pairs are graphically presented in Figure 7 and Figure 8. These ROI pairs represent the leader-follower relationships that are the most useful out of the whole lead matrix for discriminating between patients with tinnitus and controls and therefore are useful for understanding the underlying relevant cyclic functional connectivity. Figure 7 shows the flow graph where activity follows downwards and line thickness is indicative of the consistency of the relationships. It is possible to see that the normal hearing controls have much more consistent relationships, especially in regard to the activity preceding amygdalar activity. Figure 8 focuses on the graph edges where the leader-follower relationship switches direction as a function of group membership. A graphical representation of the location and networks of these ROI pairs are depicted in Figure 6.

## DISCUSSION

In this work, we have demonstrated the ability of a novel cyclicity analysis applied to resting-state functional MRI data to classify tinnitus patients and normal hearing controls. This approach not only provides a new tool the may be useful for discriminating between groups of subjects, but also provides information about the neural functional connectivity that is most helpful for discriminating between those groups.

The broadest finding, that patterns of cyclic ordering in resting-state data are helpful for classifying various groups, warrants further investigation. It has been unclear whether networks of correlated neural activity across the brain have any underlying cyclic patterns where one network or region within a network consistently precedes the activity in other networks or regions. See Keilholz (2014) for an interesting review and perspective of this question and a discussion of quasiperiodic patterns in brain activity, and Mitra, Snyder, Blazey, and Raichle (2015) for the recent demonstration of temporal sequences of propogated activity through the brain. The method used here adds to this work by incorporating a new tool capable of analyzing ordered patterns of activity that are aperiodic and vary over time. In our data, subjects showed reliable patterns of variability in their lead matrix across fMRI runs, which demonstrates the potential to analyze aspects of the cyclic signal in order to pull out subject-specific trait information. Our findings suggest that there are differential strengths of the resting signals across ROIs and differences in the consistency of the leader-follower relationships between ROI pairs. For example, in our data, the bilateral cuneus had the strongest signal in the resting-state data. However, despite the strength of the signal in these regions, it appeared that the contribution of the bilateral cuneus was similar in both the normal hearing and tinnitus groups, and was therefore likely overwhelming signals from other ROIs that were more useful in predicting the control group from the tinnitus patients.

This point may explain some of the poorer performance of the classification after dimension reduction through PCA. Much of the variation in each group’s respective lead matrix may be similar and therefore would not likely lead to good discrimination between the two groups, especially when the most consistent orderings between ROI pairs dominate the lead matrix. This may be particularly problematic for a heterogeneous condition like tinnitus, which may cause more variability in brain activity and less consistent orderings between ROI pairs. Our classification identified most of the subjects’ data as “tinnitus,” so even though there was relatively high sensitivity for identifying tinnitus through the brain data, there was very low specificity. This bias toward classifying subjects as tinnitus was ameliorated after randomly reducing the size of the tinnitus training set to the size of the control training set, although it decreased the accuracy of the classification. Thus the analysis may benefit from a larger control group in the future.

Partial least squares regression more directly analyzes the relationships between the elements of the lead matrix and the group labels (tinnitus and control). This method is therefore advantageous for discrimination because the training set is not blind to the group label in the generation of latent variables for the DA.

Another approach is to choose specific ROI pairs for further analysis as an alternative to dimension reduction through the generation of latent variables or principal components. We can use the Wilks lambda criterion to examine which ROI pairs are the most useful for discriminating between the two groups and choose a subset of those ROI pairs for further analysis.

This added benefit is useful for examining the patterns of neural activity that have some bearing on the discrimination between tinnitus patients and normal hearing subjects. In our data, we chose to use the top 20 ROI pairs given by Wilks’ lambda criterion. None of the ROI pairs by themselves has an exceptional Wilks’ lambda, but together, these 20 ROI pairs lead to better classification. The improved classification by using lambda values derived from the entire dataset to select ROI pairs for further analysis is partially due to the inherent double dipping of the data in this approach. To try to lessen the impact of double dipping in the classification, a subset of ROI pairs was selected that consistently appeared in the top 20 most discriminating lambda values when analyzing subsets of the data.

The analysis of the differences in the 20 best discriminating ROI pairs between the two groups yields the best evidence to help understand changes in neural activity that contribute to chronic tinnitus. Looking at this data, the clearest discriminating pattern of activity is that the cyclic connectivity with the amygdala is important in distinguishing tinnitus patients from controls. This result is strongly predicted from the extant literature. A review of the literature by Simonetti & Oiticica (2015) emphasizes the broad changes in neural activity across the brain in tinnitus depending on the paradigm used to study the condition. In addition, the data presented here suggests that the ordering between the amygdala and other ROIs is less constrained and more heterogeneous than that of the normal hearing controls. Davies, Gander, and Hall (2017) recently reported reduced amygdalar activity to emotionally evocative sound clips in tinnitus patients compared with controls. The heterogeneity in this region may result from differences in levels of habituation to tinnitus. Some tinnitus patients may have reduced amygdala activation following habituation, whereas others may have increased amygdala activation to the bothersome internal noise. Many nonauditory areas of the brain have been implicated in tinnitus, especially in fronto-parietal and limbic regions. Recently, Carpenter-Thompson, Schmidt, McAuley, and Husain (2015) investigated differences specifically between subgroups of patients exhibiting high distress and low distress from the condition, and found that the high distress group recruited the amygdala and parahippocampus to a greater extent in an affective sound listening task.

Changes in amgydalar activity is largely consistent with both the literature on tinnitus as well as in other conditions with some contribution of emotional dysfunction, such as depression. In depression, there is heightened amygdala responses to emotional stimuli (Sheline et al., 2001), but at the same time there is reduced connectivity between the amygdala and the affective network (Veer et al., 2010). The observed changes in cyclic connectivity may contribute to cognitive deficits in attention that have been seen in tinnitus (Trevis, McLachlan, & Wilson, 2016) and may affect the overall patterns of network connectivity that change in tinnitus (Schmidt, Akrofi, Carpenter-Thompson, & Husain, 2013; Schmidt, Carpenter-Thompson, & Husain, 2017). A loss of connectivity may also correspond with the less constrained pattern of leader-follower relationships involving the amygdala, and future research should seek to better understand this correspondence.

The classification itself is an important contribution of this work. This study serves as a proof-of-concept for the usefulness of cyclicity analysis in classifying a group of tinnitus patients from controls. The classification presented here is promising, especially for having been completed on a somewhat small sample of tinnitus patients and normal controls. Neural correlates of tinnitus would be useful for future predictions about the underlying causes of the condition, as well as leading to an objective diagnostic tool that may be validating for patients and helpful for clinicians. However, there are limits to the classification here that are evident in our analysis. First, the classification would likely benefit from a larger control group to match the sample size of the tinnitus group. Secondly, the classification after selecting ROI pairs with Wilks’ lambda are more promising, but may not extend to other samples.

Future work should apply this approach to a larger sample, which may improve classification, and compare the classification from the cyclicity analysis of resting state fMRI data to the classification from more traditional dynamic and static functional connectivity analyses. In addition, these parameters, along with behavioral and anatomical characteristics, should be combined to see if additional features contribute to even greater classification accuracy. Finally, future work will compare tinnitus patients with controls who have matched hearing loss. This is important because hearing loss often accompanies tinnitus, and it is critical that the condition of tinnitus be discriminable from other coexisting conditions. In the data presented here, most control participants have normal hearing, while the tinnitus patients do not; the differences in cyclicity patterns and promising classification could therefore be related to the differences in hearing thresholds. Alternatively accounting for hearing loss may further improve classification.

### Conclusion

Our study demonstrates that normal hearing controls and tinnitus patients can be classified through cyclicity analysis of resting-state fMRI data. Based on discriminative features (in this case, the leader-follower relationships of ROI pairs), a classification model can be built to predict if an individual has chronic tinnitus. These discriminative ROI pairs can also be used to characterize the nature of differences in brain activity between normal hearing controls and tinnitus patients, which may lead to better diagnostic tools and an improved understanding of the neural underpinnings of tinnitus. Future work will combine this cyclicity analysis with other forms of potentially discriminating data to make improved classifications of tinnitus subjects from controls.

## MATERIALS AND METHODS

### Participants

Participants were recruited from the Champaign-Urbana area as part of a larger ongoing study with community advertisements in flyers, bulletins, and newspapers. Study approval was obtained from the University of Illinois at Urbana-Champaign and written informed consent was obtained from each participant (UIUC IRB protocol no. 15955). fMRI data were collected from two groups of participants: 15 controls with no tinnitus (mean age 46.27 ± 11.71, 10 women) and 32 patients with chronic tinnitus (mean age 51.16 ± 10.73, 14 women). Demographic details are provided in Table 5.

**Table 5.** Demographics for subject groups

	**Controls (*N* = 15, 10 female)**		**Tinnitus (*N* = 32, 14 female)**		***p*-value**
	***M***	***SD***	***M***	***SD***	
Age	46.27	11.71	53.16	10.73	0.05
Beck’s Depression Inventory	3.07	6.37	4.63	5.52	0.39
Beck Anxiety Inventory	1.59	1.91	2.69	3.77	0.30

**Tinnitus Functional Index**					
Total	N/A	N/A	23.44	17.78	N/A
Intrusive	N/A	N/A	39.43	20.46	N/A
Sense of control	N/A	N/A	36.77	23.01	N/A
Cognitive	N/A	N/A	22.29	19.62	N/A
Sleep	N/A	N/A	15.99	21.36	N/A
Auditory	N/A	N/A	25.89	27.13	N/A
Relaxation	N/A	N/A	26.51	24.27	N/A
Quality of life	N/A	N/A	13.87	18.31	N/A
Emotional	N/A	N/A	9.95	10.49	N/A

**Pure tone averages**					
Right 250 Hz	12.00	10.14	13.28	6.04	0.59
Right 500 Hz	10.67	5.31	12.81	6.95	0.30
Right 1000 Hz	11.33	8.12	12.34	4.40	0.58
Right 2000 Hz	12.00	7.51	17.81	10.31	0.06
Right 3000 Hz	13.67	11.41	24.22	17.37	0.04*
Right 4000 Hz	16.00	16.50	27.03	18.62	0.06
Right 6000 Hz	16.67	19.24	30.78	19.06	0.02*
Right 8000 Hz	15.67	22.75	30.47	20.96	0.03*
Right 9000 Hz	21.00	19.29	38.75	21.96	0.01*
Right 10000 Hz	21.67	22.57	41.09	22.78	0.01*
Right 11200 Hz	30.00	25.64	47.50	21.02	0.02*
Right 12500 Hz	38.33	26.70	56.88	19.58	0.01*
Right 14000 Hz	45.33	28.19	64.22	15.35	0.00*
Right 16000 Hz	44.00	16.39	49.84	13.94	0.21
Left 250 Hz	9.00	7.37	15.00	12.25	0.09
Left 500 Hz	10.00	5.35	13.75	10.78	0.21
Left 1,000 Hz	10.00	6.55	12.50	9.67	0.37
Left 2,000 Hz	12.00	8.41	18.59	11.59	0.06
Left 3,000 Hz	15.00	12.54	27.19	16.51	0.01*
Left 4,000 Hz	17.33	18.89	29.22	17.19	0.04*
Left 6,000 Hz	17.67	17.51	34.38	18.65	0.01*
Left 8,000 Hz	14.67	16.31	33.44	21.27	0.00*
Left 9,000 Hz	17.33	15.45	43.44	22.23	0.00*
Left 10,000 Hz	19.67	15.86	47.03	22.93	0.00*
Left 11,200 Hz	25.33	22.08	52.81	23.21	0.00*
Left 12,500 Hz	37.67	27.31	60.63	23.38	0.00*
Left 14,000 Hz	46.00	29.95	63.75	18.14	0.02*
Left 16,000 Hz	41.00	20.02	50.63	14.85	0.07

* Significant at the *p* < .05 level. Scores from Becks Depression Inventory, Beck Anxiety Inventory, and the Tinnitus Functional Index were acquired at each imaging session and averaged together. Means (*M*) and standard deviations (*SD*) are presented for each group, and *p* values associated with two-sample *t* tests between group means are displayed. Pure tone averages at 250, 500, 1,000, 2,000, 3,000, 4,000, 6,000, 8,000, 9,000, 10,000, 11,200, 12,500, 14,000, and 16,000 Hz are presented for both right and left ears.

### Imaging

**Figure 9.**
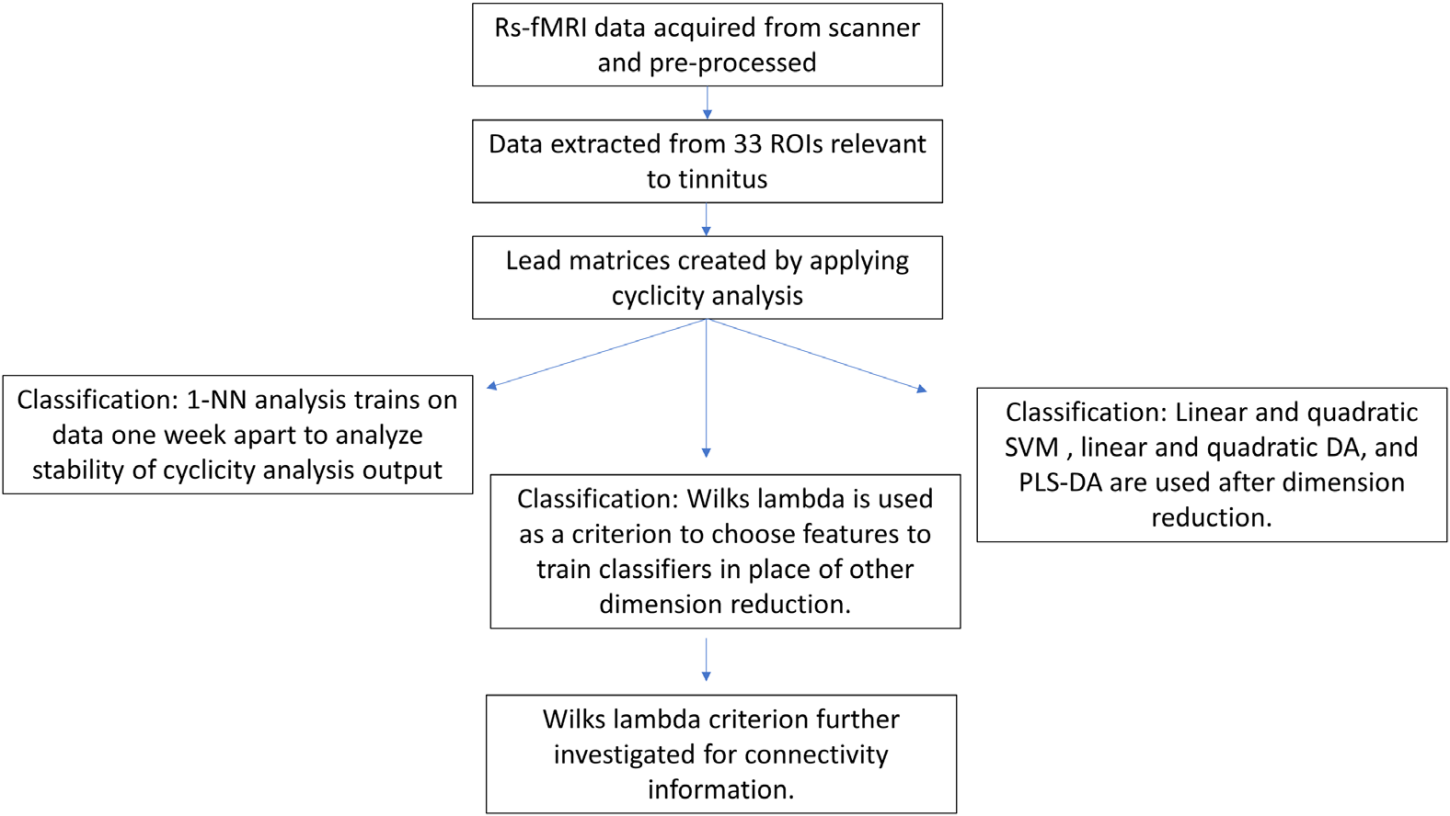
Schematic of the analysis steps. Resting-state fMRI time-course data is collected, pre-processed, and averaged in relevant ROIs. Then the cyclicity analysis is completed on the data to extract subject lead matrices. These lead matrices were used for three different types of classification. The nearest neighbor algorithm reveals the strength of the stability of a subject’s lead matrix. Classification methods are attempted after dimension reduction with PCA or through PLS-DA with 20 features. The Wilks’ lambda is used as a criterion to preselect ROI pairs to use in classification. These ROI pairs that help distinguish tinnitus subjects from controls are further investigated.

The steps involved in the imaging data analysis are described visually in Figure 9.

### Data Acquisition

All imaging data were collected using a 3T Siemens Magnetom Prisma MRI scanner. A high-resolution, T1-weighted sagittal MPRAGE image (TR = 2,300 ms, TE = 2.32 ms, flip angle = 89°, 192 slices, voxel size = 0.9 0.9 0.9 mm^3^) and a lower-resolution, T2-weighted, image (TR = 3400 ms, TE = 65 ms, flip angle = 120°, 38 slices, voxel size = 1.2 1.2 3.0 mm^3^) were both collected for use during preprocessing. Resting-state data was collected at two sessions, 1 week apart. Two 10-min runs of resting-state data were acquired at both sessions. Resting-state BOLD acquisition used a gradient echo-planar EPI sequence with transversal orientation (TR = 2,000 ms, TE = 25 ms, flip angle = 90°, 38 slices, voxel size = 2.5 2.5 3.0 mm^3^). During the resting-state scans, participants were instructed to lie still with eyes open fixated on a cross presented to them, and to not think about anything in particular. The first four volumes of each run were discarded prior to preprocessing to allow for magnet stabilization. Thus, of the 304 volumes collected in each run (four runs were collected per subject), 300 were used for subsequent analysis.

### Resting-State Preprocessing

Preprocessing was performed using SPM12 (Wellcome Trust Centre for Neuroimaging, http://www.fil.ion.ucl.ac.uk/spm/software/spm12/). Slice-time correction was first applied to the interleaved, ascending data. Functional images were realigned according to a six-parameter rigid body transformation to correct for head motion. Seven subjects were removed from subsequent analysis due to motion exceeding a 2-mm translation or 2° rotation in one of the resting-state runs. Following this, two coregistration steps were performed. First, the T2-weighted image was registered to the mean functional image generated during realignment. Second, the MPRAGE image was registered to the resulting T2-weighted image. Next, the MPRAGE image was normalized to MNI space via a nonlinear warp transformation. The resulting image was used to normalize the realigned functional data. Lastly, the functional images were smoothed using a Gaussian kernel of 8 × 8 × 8 mm^3^ full width at half-maximum.

### Regions of Interest Preparation

With the preprocessing steps completed, the resulting image files were converted into matrices to extract time courses within an ROI. From the full brain data, we selected ROIs in the brain that were to be representative of resting-state networks that have been shown to be altered in tinnitus patients. A list of ROIs, including the networks they represent, is included in Table 6. To account for the variability of location of the ROIs different subjects, we averaged the fMRI data in a region of radius 8 mm about the point listed in Table 6, using the MARSBAR toolbox in SPM (Brett, Anton, Valabregue, & Poline, 2002) to define the ROI coordinates. We chose to focus on four main networks comprising of 33 ROIs for this analysis, which have been previously implicated as differing in patients with tinnitus (Burton et al., 2012; Husain & Schmidt, 2014; Maudoux et al., 2012a, 2012b; Schmidt et al., 2013, 2017; Wineland, Burton, & Piccirillo, 2012): the default mode network, the dorsal attention network, the auditory network and limbic regions. The ROI extraction resulted in a 33 × 300 matrix as a time course per subject.

**Table 6.** Regions-of-interest used in cyclicity analysis

**Name**	**Center coordinates**	**Network**
L amygdala	−17, −2, −24	Limbic
L anterior insula	−36, 3, 7	Attention control
L cuneus	−4, −88, 16	Visual
L frontal eye field	−25, −11, 54	Dorsal attention
L inferior frontal lobe	−41, 6, 10	Attention control
L inferior parietal lobe	−31, 68, 32	Default mode
L mid frontal gyrus	−39, 11, 38	Attention control
L parahippocampus	−24, −22, −24	Limbic
L posterior intraparietal sulcus	26, −62, 53	Dorsal attention
L primary auditory cortex	55, −27, 9	Auditory
L primary visual cortex	−11, −84, 1	Visual
L superior occipital lobe	−12, −80, 23	Visual
L superior temporal junction	−49, −53, 28	Ventral attention
L superior temporal sulcus	−56, −52, 9	Ventral attention
L ventral intraparietal sulcus	−30, −83, 13	Dorsal attention
Medial prefrontal cortex	8, 59, 19	Default mode
Posterior cingulate cortex	−2, −50, 25	Default mode
Precuneus	0, −56, 50	Default mode
R amygdala	18, −7, −17	Limbic
R anterior insula	36, 3, 7	Attention control
R cuneus	4, −88, 16	Visual
R frontal eye field	27, 11, 54	Dorsal attention
R inferior frontal lobe	45, −4, 13	Attention control
R inferior parietal lobe	40, −67, 32	Default mode
R mid frontal gyrus	39, 11, 38	Attention control
R parahippocampus	23, −21, −20	Limbic
R posterior intraparietal sulcus	−23, −70, 46	Dorsal attention
R primary auditory cortex	−41, −27, 6	Auditory
R primary visual cortex	11, −84, 1	Visual
R superior occipital lobe	15, −79, 23	Visual
R superior temporal junction	49, −53, 28	Ventral attention
R superior temporal sulcus	56, −52, 9	Ventral attention
R ventral intraparietal sulcus	30, −83, 13	Dorsal attention

*Note*. ROIs used in the cyclicity analysis are listed alphabetically. L and R designate left and right regions. The center coordinates of the ROI spheres are listed asMNI (x, y, z) coordinates. The primary network membership of the region is presented.

### Cyclicity Analysis

Cyclicity analysis was performed on each time-course to generate a 33 × 33 skew-symmetric matrix (called the lead matrix), which corresponds to a 528 dimensional vector. A signal is considered cyclic if the values it takes repeat over time. A periodic signal is necessarily cyclic, but a cyclic signal need not be periodic. However, a cyclic signal can be made periodic by an appropriate time re-parameterization. Topological mathematical tools which utilize re-parameterization invariant features of paths and path spaces can be used to analyze cyclic signals by simply interpreting signals as paths (Baryshnikov & Schlafly, 2016).

To fix notation let ℝ denote the real line and ℝ^*n*^ the set of all *n*-tuples whose elements are real numbers. Let *X*_*t*_ = *X* (*u*) be a *d*-dimensional path in ℝ^*d*^ defined over an interval [*s*, *t*]. For any such path it is possible to define its *n*-th iterated integral (see K.-T. Chen, 1958) as,

$$X_{s,t}^{n}=\overset{}{\underset{s<u_{1}<\ldots <u_{2}<t}{\int }}dX_{u_{1}}⊗dX_{u_{2}}⊗\ldots ⊗dX_{u_{n}}$$ (1)

where ⊗ denotes the tensor product. Though this short-hand notation for iterated integrals is standard in literature it is important to note that *u*_*i*_ are simply integration variables. For example the first-order iterated integral is simply the increment of the path over [*s*, *t*]

$$X_{s,t}^{1}=\overset{t}{\underset{s<u_{1}<t}{\int }}dX_{u_{1}}=\overset{t}{\underset{s}{\int }}dX\left(u_{1}\right)du_{1}=X\left(t\right)-X\left(s\right)$$ (2)

and the second-order iterated integral is:

$$X_{s,t}^{2}=\overset{}{\underset{s<u_{1}<u_{2}<t}{\int }}dX_{u_{1}}⊗dX_{u_{2}}=\overset{t}{\underset{s}{\int }}\overset{u_{2}}{\underset{s}{\int }}dX_{u_{1}}⊗dX_{u_{2}}$$ (3)

With the definition of the zeroth iterated integral to be 1 the infinite collection of iterated integrals (**X**_*s*,*t*_^0^, **X**_*s*,*t*_^1^, **X**_*s*,*t*_^2^, …) is called the signature of a path (Chevyrev & Kormilitzin, 2016). The signature of a path defines many of its algebraic and geometric properties, and is invariant under translations as well as re-parameterizations (K.-T. Chen, 1971). Then it is possible to consider the time-course data from each run per subject as a path and construct its *truncated* signature as an object of interest.

For our purposes, the second-order iterated integral of the closed path (we mean-center and linearly adjust the time course so that it becomes a closed path) gives us meaningful information about the relationships between pairs of ROIs. Each row and column of the constructed *lead matrix*

$$A:=\frac{1}{2}\overset{}{\underset{s<u_{1}<u_{2}<t}{\int }}dX_{u_{1}}⊗dX_{u_{2}}-dX_{u_{2}}⊗dX_{u_{1}}$$ (4)

can be associated with an ROI, and specifically, each element of the lead matrix corresponds to the signed algebraic area obtained by projecting the *n*-dimensional path down to a two-coordinate plane (the coordinates being the two ROIs). When this area is positive, the signal in the second ROI (column) can be considered to follow the first ROI (row). Note that the lead matrix for a path *X* is simply the antisymmetric component of **X**^2^. Spectral analysis of the lead matrix then allows for the extraction of other features from the dataset. Specifically we can attribute a *strength* to the signal from a particular ROI, as well as extract an estimated ordering for the *cycle* among all the ROIs (Baryshnikov & Schlafly, 2016).

**Figure 10.**
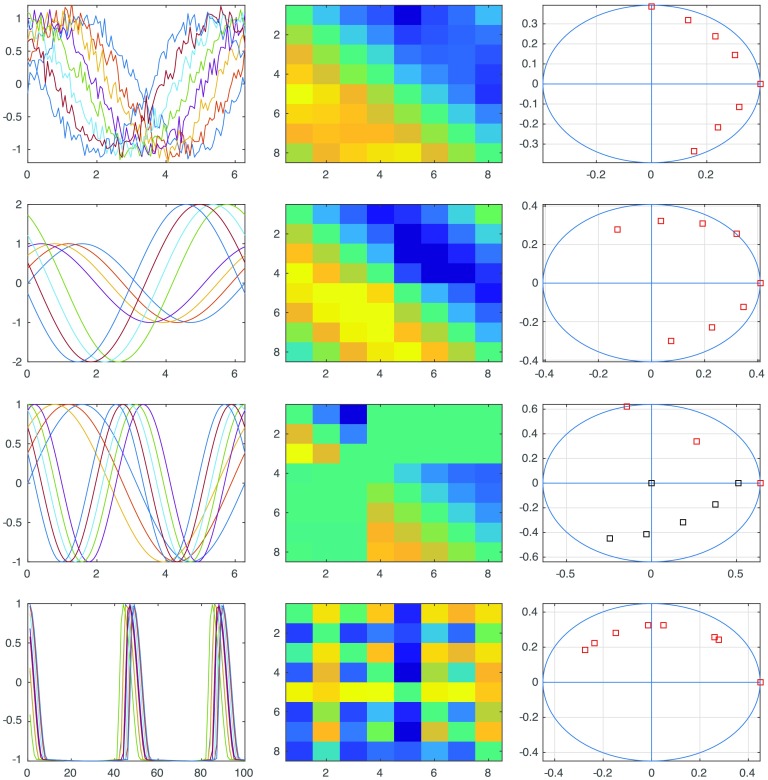
Panel showing how to interpret the lead matrix and phase components. First row shows eight phase-shifted sinusoids that have added white noise with SNR = 20, the lead matrix obtained from it as well as approximate phase offsets recovered from the first principal eigenvector. Second row shows two set of sinusoids in a similar fashion, but with one set having different magnitude and being offset from the other set. Third row shows a signal containing two harmonics, which requires the use of two principal eigenvectors to recover phases completely; note how the lead matrix has an added zero component (the second harmonic is twice the first) giving a point at the origin. The final set contains a traditional cyclic but aperiodic signal and its corresponding results.

A few simulated and simple cases are shown in Figure 10. In the case of a path consisting of a single harmonic that is phase shifted, near perfect recovery of the shifts is possible even in the presence of noise, as shown in the first example. The second example demonstrates that with appropriate mean centering and normalization two sets of signals with offsets between them, as well as among the sets themselves can be analyzed to approximately recover the phase offsets. The third example shows how the analysis leads to more involved spectral decomposition of the lead matrix when more than one harmonic is present in the signal. Finally, the last example shows how phase recovery is possible even when the signals themselves are merely cyclic and aperiodic.

Although the first three simulated examples above are trivially solved by Fourier theory, the cylicity methods extends to cyclic but aperiodic signals. The last example can also be analyzed by methods involving lagged time correlation (Mitra et al., 2015) but the benefit of cyclicity analysis is that the theoretical underpinnings are re-parameterization invariant with respect to time, that is, **X**_*t*_ → **X**_*ϕ*(*t*)_. This allows one to work under the hypothesis that even though the underlying generative process maybe the same, the time scales involved maybe different.

A preliminary version of the cyclicity analysis toolbox is available online at: <http://cycapp.herokuapp.com> and more details can be found at: <http://acnlab.beckman.illinois.edu/#/>

#### Stability of cyclicity analysis.

To assess the stability of a subject’s lead matrix across the two sessions, separated by 1 week, we used a *k*-NN classifier (*k* = 1, cosine metric) to train on the subject’s session 1 data and test on session 2 data, and then train on session 2 data and test on session 1 data. Subjects were not separated by group for this analysis. Therefore, each subject could be correctly classified as themselves four times, amongst all of the subjects involved in the study. A high classification rate would mean that the high similarity between the feature space from the subjects lead matrices in one session was sufficient to predict who the subject was in the alternative session.

### Classification

Lead matrices were used to classify tinnitus patients from controls following two procedures of dimension reduction. In the first, PCA was used to generate orthogonal components from the lead matrix. The top 10 components were kept for subsequent classification. In the second case, Wilks’ lambda values on each of the lead matrix features (ROI pairs) was used to select the most discriminating features for subsequent classification. Classification analyses were performed in Matlab by using the Classification Learner App as well as by using a Classification Toolbox (Ballabio & Consonni, 2013) for the PLS-DA method.

#### Classification after dimension reduction.

Dimensionality reduction to 10 dimensions was achieved with PCA to prepare the data for quadratic and linear classification methods. Four methods were chosen for classification: a linear SVM, quadratic SVM, linear DA, and quadratic DA. In addition, 20 component PLS-DA was also implemented as a classification method. PLS regression utilizes the class label information to choose components that best explain the correlation between X and Y (the vector of class labels). In PCA when used as a dimension reduction technique, classification is performed using components generated from the data itself. Therefore, PCA is an excellent method for extracting uncorrelated features in the data, but does not guarantee that these features have discriminatory value in the classification problem. In contrast, in PLS the class information of the training set is used to generate the latent variables that are specifically useful for discrimination. Half of the tinnitus patients and half of the controls were randomly chosen to be part of the training set, and half comprised the test set.

#### Classification on ROI pairs chosen using Wilks’ lambda.

The Wilks’ lambda criterion can be used to help select the most discriminatory elements in the lead matrix. Lambda values were computed on the whole dataset, but used to inform the selection of variables for the classifier when training on half the dataset and testing on the other half. Since lambda values may be specific to this particular sample, a subset of ROI pairs were further chosen according to which ROI pairs were the most stable across subsets. One thousand Monte Carlo trials were run on half of the dataset to determine which ROI pairs most consistently showed the lowest 20 lambda scores (and therefore had the most stable discriminating power). The top 10 most stable ROI pairs were chosen from this analysis for subsequent classification. The classification rate was based on the average of 100 Monte Carlo trials when training on half of the dataset and testing on the other half.

To provide insights into what functional activity is changing between patients with tinnitus and controls, the 20 ROI pairs with the most discriminating power were further examined. Graphs were made using ROIs corresponding to the most discriminatory features from Wilks’ lambda criterion, using the ROIs as nodes and group average value of the feature (from the normalized lead matrix to account for individual differences) as edge weights. To consider the proportion of a group exhibiting a certain directionality one can, instead of normalizing the lead matrix, also set its elements to ± 1 depending on direction and observe which relations are flipped at the group level.

## ACKNOWLEDGMENTS

We would like to acknowledge Beckman Institute Biomedical Imaging Center staff for their help in data acquisition and to thank Yihsin Tai, AuD, and Anthony Tsao, AuD, for assistance with audiological assessments.

## AUTHOR CONTRIBUTIONS

Benjamin J. Zimmerman: Conceptualization; Formal analysis; Investigation; Methodology; Writing – original draft; Writing – review & editing. Ivan Thomas Abraham: Conceptualization; Formal analysis; Methodology; Writing – original draft; Writing – review & editing. Sara Ann Schmidt: Investigation; Methodology; Writing – original draft; Writing – review & editing. Yuliy Baryshnikov: Conceptualization; Investigation; Methodology; Supervision. Fatima T. Husain: Conceptualization; Funding acquisition; Investigation; Methodology; Project administration; Supervision; Writing – original draft; Writing – review & editing.

## FUNDING INFORMATION

Fatima T. Husain, Defense Health Agency (http://dx.doi.org/10.13039/100009898), Award ID: W81XWH-15-2-0032.

## TECHNICAL TERMS

Cyclic signal: A cyclic signal is one that repeats itself identically over and over again but perhaps with a variable speed. Periodic signals are cyclic while converse is not true.
Phase magnitudes: Each complex entry of an eigenvector of the lead matrix corresponds to an ROI. The phase magnitudes associated with an eigenvalue *λ* for each ROI are the absolute values of these entries.
Cosine metric: A measure of similarity, 1 − cos *θ*, between real valued finite dimensional vectors *u*, *v*, defined using the dot product *u* ⋅ *v* = ∥*u*∥∥*v*∥ cos *θ*. It is zero if and only if one vector is a positive multiple of the other; it is not a true metric.
Wilks’ lambda: A measure of discriminatory ability of a variable, defined by $\Lambda =\frac{\left|A\right|}{\left|A+B\right|}$ where *A* is the within group sum of squares and cross products matrix (SSCP), *B* is the between group SSCP matrix and |⋅| denotes determinant.
Skew-symmetric matrix: A real matrix *A* is skew symmetric if *a*_*kl*_ = −*a*_*lk*_. Such a matrix has eigenvalues that are zero or purely imaginary complex conjugates with corresponding eigenvectors in complex conjugate pairs.
Periodic signal: A signal or time series is *periodic* if the shift of time ahead by a value *P* keeps the series unchanged. The smallest such *P* is called its period.
Re-parameterization: A re-parameterization of a path over an interval [*s*, *t*] is a change of variable *X*(*t*) → *X*(*ϕ*(*t*)) such that *X*(*s*) = *X*(*ϕ*(*s*)), *X*(*t*) = *X*(*ϕ*(*t*)) and *ϕ*(⋅) is continuous, bounded and increasing over [*s*, *t*].
Path: An alias for a multidimensional signal, i.e., a *d* dimensional path is simply a smooth mapping from a subset of the real line to a vector space, e.g., *X* : [*s*, *t*] ⊂ ℝ → ℝ^*d*^.
Tensor product ⊗: For real valued finite dimensional vectors (matrices) considered in this paper it is equivalent to the outer (Kronecker) product.
